# Supplementary material for: Multi‐dataset identification of innovative feature genes and molecular mechanisms in keratoconus
Source: J Cell Mol Med. 2024 Sep 19;28(18):e70079. doi: 10.1111/jcmm.70079 (PMC11412914; doi:10.1111/jcmm.70079)
Supplement: Supplementary file 2 — Material S2. [file JCMM-28-e70079-s002.zip › 7.GSEA analysis/2.ATOH7/KEGG_THYROID_CANCER.html]

Details for gene set KEGG\_THYROID\_CANCER[GSEA]

|  || Dataset | ATOH7.group.cls#h\_versus\_l |
| Phenotype | group.cls#h\_versus\_l |
| Upregulated in class | h |
| GeneSet | KEGG\_THYROID\_CANCER |
| Enrichment Score (ES) | 0.41950926 |
| Normalized Enrichment Score (NES) | 1.4333358 |
| Nominal p-value | 0.06790123 |
| FDR q-value | 0.28580365 |
| FWER p-Value | 0.869 |
Table: GSEA Results Summary

  

Fig 1: Enrichment plot: KEGG\_THYROID\_CANCER      
 Profile of the Running ES Score & Positions of GeneSet Members on the Rank Ordered List

  

| SYMBOL | TITLE | RANK IN GENE LIST | RANK METRIC SCORE | RUNNING ES | CORE ENRICHMENT || 1 | LEF1 | na | 735 | 0.492 | 0.1284 | Yes |
| 2 | RXRG | na | 913 | 0.462 | 0.2637 | Yes |
| 3 | PPARG | na | 2303 | 0.323 | 0.3218 | Yes |
| 4 | TCF7 | na | 3059 | 0.277 | 0.3843 | Yes |
| 5 | MAP2K1 | na | 4240 | 0.228 | 0.4195 | Yes |
| 6 | TCF7L1 | na | 6644 | 0.153 | 0.3967 | No |
| 7 | NRAS | na | 7693 | 0.126 | 0.4047 | No |
| 8 | TPR | na | 10221 | 0.078 | 0.3556 | No |
| 9 | RXRB | na | 12340 | 0.048 | 0.3091 | No |
| 10 | TFG | na | 14049 | 0.030 | 0.2690 | No |
| 11 | MAPK1 | na | 14536 | 0.025 | 0.2628 | No |
| 12 | HRAS | na | 14559 | 0.025 | 0.2698 | No |
| 13 | TP53 | na | 14698 | 0.024 | 0.2730 | No |
| 14 | MAPK3 | na | 16821 | 0.006 | 0.2137 | No |
| 15 | NCOA4 | na | 18201 | -0.006 | 0.1757 | No |
| 16 | CTNNB1 | na | 18214 | -0.006 | 0.1772 | No |
| 17 | CCDC6 | na | 19434 | -0.017 | 0.1471 | No |
| 18 | MYC | na | 19651 | -0.019 | 0.1465 | No |
| 19 | CDH1 | na | 19700 | -0.019 | 0.1509 | No |
| 20 | CCND1 | na | 20546 | -0.027 | 0.1348 | No |
| 21 | MAP2K2 | na | 20653 | -0.028 | 0.1403 | No |
| 22 | TPM3 | na | 21305 | -0.035 | 0.1322 | No |
| 23 | RXRA | na | 21798 | -0.041 | 0.1304 | No |
| 24 | TCF7L2 | na | 22134 | -0.044 | 0.1342 | No |
| 25 | BRAF | na | 25048 | -0.086 | 0.0765 | No |
| 26 | KRAS | na | 25083 | -0.087 | 0.1018 | No |
| 27 | NTRK1 | na | 25343 | -0.091 | 0.1221 | No |
| 28 | RET | na | 29684 | -0.200 | 0.0578 | No |
| 29 | PAX8 | na | 31897 | -0.288 | 0.0816 | No |
Table: GSEA details [plain text format]

  

Fig 2: KEGG\_THYROID\_CANCER      
 Blue-Pink O' Gram in the Space of the Analyzed GeneSet

  

Fig 3: KEGG\_THYROID\_CANCER: Random ES distribution      
 Gene set null distribution of ES for **KEGG\_THYROID\_CANCER**
